# Reduced DNA methylation of the oxytocin receptor gene is associated with obsessive-compulsive disorder

**DOI:** 10.1186/s13148-020-00890-w

**Published:** 2020-07-06

**Authors:** Chun Il Park, Hae Won Kim, Sumoa Jeon, Jee In Kang, Se Joo Kim

**Affiliations:** 1 Department of Psychiatry, CHA Bundang Medical Center, CHA University, Seongnam, Republic of Korea; 2 Department of Psychiatry, Yonsei University College of Medicine, Yonsei-ro 50-1, Seodaemun-gu, Seoul, 03722 Republic of Korea; 3 Institute of Behavioral Science in Medicine, Yonsei University College of Medicine, Seoul, Republic of Korea; 4 Department of Medical Education, Yonsei University College of Medicine, Seoul, Republic of Korea

**Keywords:** Obsessive-compulsive disorder, Oxytocin, *OXTR*, Epigenetics, DNA methylation

## Abstract

**Background:**

Oxytocin is an important neuromodulator involved in cognition and socio-emotional processing that exerts its central activities via oxytocin receptors. Epigenetic alterations in the oxytocin receptor gene (*OXTR*) may be a molecular mechanism in the pathogenesis of obsessive-compulsive disorder (OCD). This study investigated the association between *OXTR* DNA methylation and the OCD status of a Korean population.

**Results:**

Quantitative leukocyte DNA methylation levels of three cytosine-phosphate-guanine (CpG) sites in the 5′ untranslated region (UTR) of *OXTR* exon 2 and eight CpG sites within *OXTR* exon 3 were analyzed using the pyrosequencing method in 151 patients with OCD (including 45 drug-naïve patients) and 108 healthy controls. DNA methylation levels were compared between the groups using multiple analyses of covariance separately by sex after controlling for age and educational level. Patients with OCD showed significantly lower methylation levels at CpG1 and CpG2 sites on the UTR of *OXTR* exon 2 than those of healthy controls for both sexes. In a subset of 45 drug-naïve patients with OCD, the DNA methylation levels also remained significantly lower than those in the controls and their CpG1 methylation levels were significantly negatively associated with the ordering symptom dimension.

**Conclusions:**

Our findings suggest that epigenetic *OXTR* alterations may affect the pathophysiology of OCD. The potential role of the oxytocin system in OCD development and treatment warrants further investigation.

## Background

Oxytocin is an important neuropeptide that regulates social behaviors and is involved in various cognitive and emotional processes [1, 2]. Since oxytocin exerts its central activities via oxytocin receptors (*OXTR*) in the brain, studying alterations in *OXTR* is of great importance for understanding the molecular mechanisms underlying the pathogenesis of psychiatric conditions related to socio-emotional processing [3–5]. DNA methylation, an epigenetic mechanism by which cells dynamically control gene expression without changing the DNA sequence, has emerged as a crucial mechanism underlying the interaction between genetic background and environmental factors in the development of psychiatric disorders [6]. It has been reported that differential *OXTR* DNA methylation is involved in various psychological functions relevant to psychopathology including callous-unemotional traits [7], social perception [8], and resilience [9] and is also involved in psychiatric illnesses such as autism spectrum disorder (ASD) [4], postpartum depression [10], social anxiety disorder [11], and early-stage schizophrenia [12].

Epigenetic alterations in the *OXTR* gene are a promising candidate marker for mediating genetic susceptibility to obsessive-compulsive disorder (OCD), which is characterized by recurrent and disturbing obsessions and repetitive compulsive behaviors. OCD is known to develop via complex interactions between genetic and environmental factors [13]. The oxytocin system has been implicated as having a role in the pathogenesis and treatment of OCD [14]; however, little research has been carried out on the epigenetics of *OXTR* in OCD. Studies of the oxytocin system in OCD have primarily focused on changes in oxytocin levels [15, 16] and the DNA sequence without considering the role of epigenetic factors [17]. Recently, an epigenetic study with a small sample size showed that *OXTR* hypermethylation was associated with OCD susceptibility and symptom severity [18]. Given the emerging role of *OXTR* in OCD pathogenesis, investigating epigenetic *OXTR* alterations may be important for understanding the molecular mechanisms underlying OCD pathogenesis and discovering better biomarkers for OCD development and progression.

In this study, we investigated differences in the leukocyte DNA methylation level of *OXTR* between patients with OCD and healthy controls. To exclude the potential effects of medication on DNA methylation in patients with OCD, we examined whether the results of the whole cohort were confirmed in a subgroup of drug-naïve patients with OCD. Moreover, we examined how DNA methylation levels at specific *OXTR* cytosine-phosphate-guanine (CpG) sites were associated with symptom severity and OCD dimensions.

## Results

### Demographics and clinical characteristics

The demographics and clinical features of each group are presented in Table 1. There were significant differences in the DNA methylation levels of several *OXTR1* and *OXTR2* CpG sites between men and women. Women in the control group showed higher DNA methylation levels at *OXTR1* CpG1 (*t* = − 2.40, *p* = 0.018) and CpG2 (*t* = − 2.83, *p* = 0.006). Similarly, women in the OCD group showed hypermethylation at all three *OXTR1* sites compared to the men: CpG1 (*t* = − 2.08, *p* = 0.039), CpG2 (*t* = − 2.80, *p* = 0.006), and CpG3 (*t* = − 2.27, *p* = 0.025). For *OXTR2*, there was no significant difference between women and men in either the control or patient groups.

**Table 1:** Demographics and clinical features of participants

	Men			Women		
	OCD^a^ (*n* = 112)	HC^a^ (*n* = 56)	*P*^b^	OCD^a^ (*n* = 39)	HC^a^ (*n* = 52)	*P*^b^
Age	21.9 ± 2.2	21.5 ± 3.5	0.385	21.8 ± 2.2	21.1 ± 3.6	0.269
Education, year	12.7 ± 1.7	13.5 ± 1.7	0.006	13.1 ± 1.8	13.6 ± 1.9	0.26
Age of onset	14.3 ± 4.1			14.7 ± 4.0		
Duration of illness, year	7.6 ± 4.5			7.0 ± 3.9		
MADRS	20.6 ± 9.8			19.7 ± 9.7		
Y-BOCS	25.0 ± 6.9			25.9 ± 6.5		

*OCD* obsessive-compulsive disorder, *HC* healthy control, *MADRS* Montgomery-Asberg Depression Rating Scale, *Y-BOCS* Yale-Brown Obsessive-Compulsive Scale

^a^Mean ± standard deviation

^b^Independent samples *t* test

### Group differences in OCD status due to *OXTR* DNA methylation

As shown in Table 2, multivariate analysis of covariance (MANCOVA) revealed that OCD status had a significant overall effect on DNA methylation levels in both men (Wilks *λ* = 0.822, *F*_(4, 161)_ = 8.693, *p* < 0.001) and women (Wilks *λ* = 0.736, F_(4, 84)_ = 7.525, *p* < 0.001). Post hoc comparisons using Bonferroni-adjusted alpha for the four dependent variables indicated that patients with OCD had significantly lower methylation levels at *OXTR1* CpG1 and CpG2 sites and no significant difference at *OXTR1* CpG3 compared to healthy controls for both men and women. No significant findings were found for *OXTR2* methylation levels.

**Table 2:** Results of MANCOVA of OXTR DNA methylation between patients with OCD and healthy controls for men and women

		Men					Women				
		OCD^a^ (*n* = 112)	HC^a^ (*n* = 56)				OCD^a^ (*n* = 39)	HC^a^ (*n* = 52)			
				*F*	*p*^b^	η_p_^2c^			*F*	*p*^b^	η_p_^2c^
		Statistics: Wilks *λ* = 0.822, *F*_(4, 161)_ = 8.693, *p* < 0.001					Statistics: Wilks *λ* = 0.736, *F*_(4, 84)_ = 7.525, *p* < 0.001				
*OXTR*1 (exon2)	CpG1 (-959)	40.94 ± 5.17	45.67 ± 4.88	20.012	< 0.001	0.109^†^	42.94 ± 5.13	47.70 ± 3.89	21.784	< 0.001	0.200^‡^
	CpG2 (-934)	46.33 ± 5.21	50.44 ± 4.52	22.873	< 0.001	0.122^†^	49.11 ± 5.75	52.54 ± 3.14	10.631	0.002	0.109^†^
	CpG3 (-924)	58.08 ± 5.25	59.62 ± 3.82	1.835	0.177	0.011	60.36 ± 5.86	60.74 ± 3.75	0.032	0.858	< 0.001
*OXTR*2 (exon3)	Mean value of CpG1-8	13.81 ± 5.46	13.34 ± 5.66	0.233	0.630	0.001	14.80 ± 4.49	14.14 ± 4.57	0.020	0.888	< 0.001

MANCOVA demonstrated significant between-group differences after controlling for age and education

*MANCOVA* multivariate analysis of covariance, *OCD* obsessive-compulsive disorder, *HC* healthy control

^a^Mean ± standard deviation of raw data

^b^Statistical significance was set at *p* < 0.0125 after Bonferroni correction for the 4 CpG sites

^c^Effect size was calculated using partial eta squared and interpreted according to the rule of Miles and Shevlin (2001)

^†^Medium effect (> 0.06)

^‡^Large effect (> 0.14)

When the same analysis was carried out on a subset of drug-naïve subjects to exclude the potential effects of medication on DNA methylation levels, the significant *OXTR* findings were still significant in both sexes. MANCOVA revealed significant overall effects on the affective status of OCD in men (Wilks *λ* = 0.840, *F*_(4, 81)_ = 3.843, *p* = 0.007) and women (Wilks *λ* = 0.666, *F*_(4, 58)_ = 7.285, *p* < 0.001) in *OXTR1*, but not in *OXTR2*. Drug-naïve patients with OCD showed significantly lower levels of DNA methylation at CpG1 (men: *F* = 7.642, *p* = 0.007, η_p_^2^ = 0.083; women: *F* = 10.389, *p* < 0.001, η_p_^2^ = 0.193) and CpG2 (men: *F* = 14.633, *p* < 0.001, η_p_^2^ = 0.148; women: *F* = 5.908, *p* = 0.002, η_p_^2^ = 0.147) sites than the healthy controls in both sexes (Table 3). There was no significant difference in the DNA methylation status between drug-naïve and drug-treated patients with OCD for all the CpG sites in each sex (Supplementary Table S1).

**Table 3:** Results of MANCOVA of DNA methylation at *OXTR* CpG sites between drug-naïve patients with OCD and healthy controls for men and women

		Men					Women				
		OCD^a^ (*n* = 32)	HC^a^ (*n* = 56)				OCD^a^ (*n* = 13)	HC^a^ (*n* = 52)			
				*F*	*p*^b^	η_p_^2c^			*F*	*p*^b^	η_p_^2c^
		Statistics: Wilks *λ* = 0.840, *F*_(4, 81)_ = 3.843, *p* = 0.007					Statistics: Wilks *λ* = 0.666, *F*_(4, 58)_ = 7.285, *p* < 0.001				
*OXTR*1 (exon2)	CpG1 (-959)	41.49 ± 5.66	45.67 ± 4.88	7.642	0.007	0.083^†^	42.75 ± 5.96	47.70 ± 3.89	10.389	< 0.001	0.193^‡^
	CpG2 (-934)	46.41 ± 4.73	50.44 ± 4.52	14.633	< 0.001	0.148^‡^	48.87 ± 3.72	52.54 ± 3.14	5.908	0.002	0.147^‡^
	CpG3 (-924)	57.41 ± 4.45	59.62 ± 3.82	6.044	0.016	0.067	61.49 ± 6.12	60.74 ± 3.75	0.695	0.368	0.013
*OXTR*2 (exon3)	Mean value of CpG1-8	14.32 ± 6.31	13.34 ± 5.66	0.388	0.535	0.005	13.79 ± 4.27	14.14 ± 4.57	0.274	0.599	0.005

MANCOVA demonstrated significant between-group difference after controlling for age and education

*MANCOVA* multivariate analysis of covariance, *OCD* obsessive-compulsive disorder, *HC* healthy control

^a^Mean ± standard deviation of raw data

^b^Statistical significance was set at *p* < 0.0125 after Bonferroni correction for 4 CpG sites

^c^Effect size was calculated using partial eta squared and interpreted according to the rule of Miles and Shevlin (2001)

^†^Medium effect (> 0.06)

^‡^Large effect (> 0.14)

### Relationships between *OXTR* methylation and clinical characteristics of OCD

**Fig. 1:**
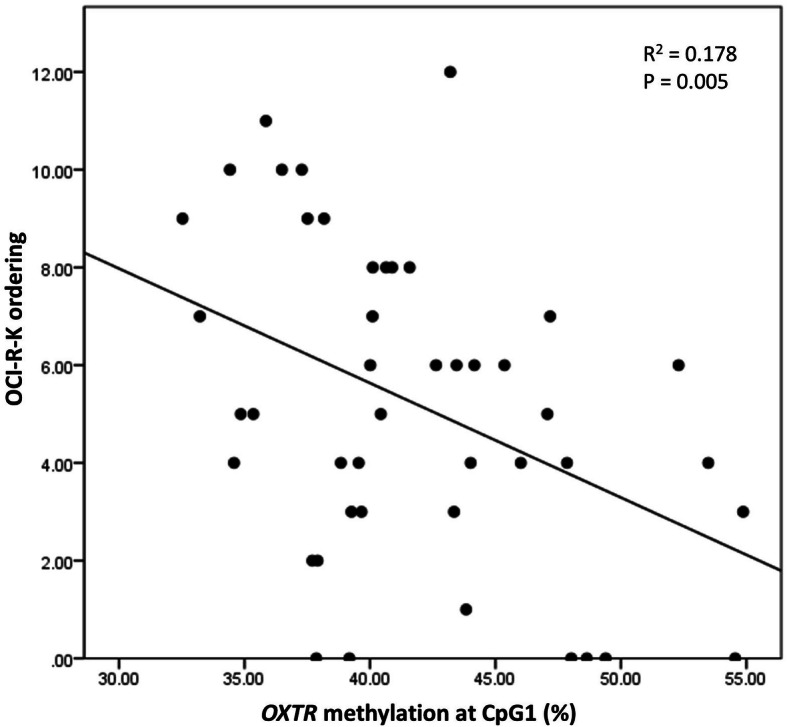
Correlation between DNA methylation levels at *OXTR1* CpG1 and ordering symptom severity in drug-naïve patients with OCD (*n* = 45). *OXTR*, oxytocin receptor gene; OCI-R-K, obsessive-compulsive inventory-revised-Korean; OCD, obsessive-compulsive disorder

Partial correlation analyses among the drug-naïve patients with OCD revealed that OCD symptom severity measured by Yale-Brown Obsessive Compulsive Scale (Y-BOCS) had no significant association with DNA methylation levels at *OXTR1* CpG1 and CpG2, which showed a significant between-group difference (*r* = 0.146 and *p* = 0.358 for CpG1; *r* = -0.211 and *p* = 0.18 for CpG2). Among the six OC symptom dimensions (washing, obsessing, hoarding, ordering, checking, and neutralizing) measured by the Korean version of Obsessive-Compulsive Inventory-Revised (OCI-R-K), ordering dimension scores were significantly and negatively correlated with DNA methylation at the CpG1 site (*r* = − 0.422, *p* = 0.005; Fig. 1). Conversely, CpG1 DNA methylation levels were not significantly associated with any OCD dimensions.

## Discussion

The role of epigenetic OXTR regulation via DNA methylation in psychiatric disorders such as OCD is a promising and relatively new field of investigation. The key finding of this study was that both male and female patients with OCD showed significantly altered *OXTR1* methylation status. These significant findings were also confirmed in a subset of drug-naïve patients with OCD, suggesting that *OXTR1* epigenetic alterations may play a crucial role in the pathogenesis of OCD and act as a molecular mechanism underlying the development and recovery of OCD, possibly via interactions with genetic predisposition and environmental factors.

In this study, patients with OCD exhibited DNA hypomethylation (which may indicate higher *OXTR* expression) at CpG1 (Chr3: 8,810,833) and CpG2 (Chr3: 8,810,808) in the 5′ untranslated region (*UTR*) of *OXTR* exon 2 compared to healthy controls. To date, only one study with a small sample size (42 patients with OCD vs. 31 controls) has reported *OXTR* methylation in OCD, finding that hypermethylation of specific CpG sites in *OXTR* exon 3 was associated with OCD disease status, correlating positively with OC symptom severity and negatively with depressive scores [18]. Although various differences between our study and theirs, including age and target CpG sites, make direct comparison difficult, their findings indicated that the associations of *OXTR* methylation acted in the opposite direction to our findings. Compared to the previous study, we believe that our study has several advantages, including a larger sample size and a more reliable OCD recruitment method (tertiary hospital vs. advertisement), and our findings showed consistent results in both male and female subjects as well as in drug-naïve patients. Furthermore, several studies have reported similar findings from fields of psychiatry other than OCD; for instance, the same direction of *OXTR* hypomethylation was observed in a clinical study of ASD [19]. A recent study of early-stage schizophrenia also demonstrated that hypomethylation status at the same genomic *OXTR* position as our significant results was associated with susceptibility to schizophrenia and anhedonia-asociality in women [12]. A systematic narrative review of *OXTR* DNA methylation in human socio-emotional functioning suggested that while *OXTR* hypermethylation may play a role in the general impairment of socio-emotional functioning, *OXTR* hypomethylation may play a role in specific patterns of impairment related to psychiatric disorders, such as anxiety disorders [3]. As proposed by Ziegler et al. who demonstrated the relationship between *OXTR exon 3* hypomethylation and social anxiety, *OXTR* hypomethylation *findings* suggest that higher *OXTR* expression might act as compensatory upregulation for reduced oxytocin levels [11]. However, previous studies on OCD could not conclude that altered oxytocin level in cerebrospinal fluid was associated with disease status [15, 20]. Interestingly, Marroni et al. argued that the oxytocin receptor’s failure to respond to oxytocin activation might be involved in the continuation of compulsive behavior in their study which investigated an experimental animal model of hypergrooming behavior [21]. In addition, some basic studies suggested that inhibited neuronal activity induced DNA demethylation of specific receptors [22, 23]. The relationship between methylation status and dysfunctional receptor properties or signaling in oxytocin system is still unknown, and it may be an alternative area to investigate. Therefore, it is crucial to elucidate the mechanisms of interaction between *OXTR* methylation and oxytocin receptor dysfunction in patients with OCD in future studies. Since there is currently very limited evidence on epigenetic *OXTR* regulation in psychiatric fields including OCD and the direction of the association has been reported to show mixed findings, further longitudinal studies or preclinical studies are required to better establish the role of *OXTR* methylation in OCD pathogenesis to determine whether altered *OXTR* methylation status confers vulnerability to OCD development or is a consequence of OCD.

Given the distinct neural correlates of OC symptom dimensions [24], we examined whether the DNA methylation levels at the *OXTR1* CpG sites that showed a significant between group-difference were associated with certain symptom dimensions in drug-naïve patients with OCD. Among the six symptom dimensions assessed by the OCI-R-K, DNA methylation levels at *OXTR1* CpG1 were found to be inversely associated with ordering dimension scores in drug-naïve patients with OCD [25–27]. Patients with high ordering dimension scores, characterized by ordering and arranging their surroundings and ensuring that objects are arranged in exactly the right way, have been found to have different characteristics, such as an earlier onset age, a more familial OCD form, impaired set-switching abilities, and a dissociable neural system [24, 28, 29]. Epigenetic *OXTR* regulation may be a molecular mechanism underlying the clinical and biological differences in the ordering dimension. Although it is unclear how *OXTR* methylation relates specific to the symptom dimension, it is possible that the ordering/arranging symptom dimension includes shared clinical features of OCD and ASD [25–27] that might be modulated by *OXTR* methylation. This will need to be elucidated in further studies.

This study has several limitations to consider. Firstly, since we cannot measure the methylation status of human brain tissue directly in a clinical study, we assumed that the DNA methylation status of the peripheral blood reflected that of the CNS. Although altered *OXTR* DNA methylation in the blood has been shown to be significantly associated with gene expression in brain tissue [4] and the neural response of social and emotional processing [8, 30, 31], their relationship across tissues has not yet been clearly established. Secondly, although *OXTR* methylation status have been observed to be associated with *OXTR* mRNA expression [4], hormone analysis of circulating oxytocin levels and mRNA expression of oxytocin and *OXTR* were not evaluated in this study. Thus, we cannot determine the functional significance of the CpGs studied and their relationship with oxytocin levels. Thirdly, since different genotypes may affect DNA methylation, we could not completely rule out the potential effect of different genotypes of *OXTR* gene on the present results. However, we previously reported that there was no significant difference in the genotype distributions and the haplotype frequencies for 10 common SNPs (rs1042778, rs237885, rs237887, rs2268490, rs4686301, rs2268493, rs2254298, rs13316193, rs53576, and rs2268498) on *OXTR* gene between patients with OCD (*N* = 615) and healthy controls (*N* = 581) [17]. When we additionally examined in a subset of the present sample with OCD (*n* = 129) whether the different genotypes of the 10 SNPs of *OXTR* gene have different methylation levels of *OXTR1*, there was no association between *OXTR* SNPs and methylation levels in the CpGs of *OXTR1* (all *p* > 0.05). Fourthly, we did not consider potential confounders that may affect the DNA methylation status of the *OXTR* gene, such as cigarette smoking status [32], heterogeneity of white blood cell types [33], and exposure to environmental factors such as diet, maternal care, and lifestyle [34]. Hence, further research that considers these potential confounders and gene-environment interactions are warranted to confirm our results. Fifthly, we cannot conclude whether the epigenetic *OXTR* alterations reflect specific OCD factors or shared traits with other psychiatric disorders related to socio-emotional problems and repetitive behaviors. Lastly, our study had a cross-sectional design; thus, conclusions cannot be drawn regarding the causal direction of the relationships between epigenetic alterations and OCD. To better establish the role of *OXTR* methylation and causal relationships in the pathogenesis and course of OCD, further longitudinal studies are needed.

## Conclusions

The present study showed altered DNA methylation status of *OXTR1* in both male and female patients with OCD as well as in a subset of drug-naïve OCD patients, compared to healthy controls. Our findings suggest that epigenetic alterations of *OXTR* may exert an effect on the pathophysiology of OCD. The potential role of *OXTR* in the development and treatment of OCD warrants further investigation.

## Methods

### Participants

A total of 151 patients (112 men, 39 women) with OCD were recruited from a specialized OCD outpatient clinic at Severance Hospital of Yonsei University Health System (Seoul, Republic of Korea), a tertiary care hospital. All patients were referred from primary care for OCD and were assessed by trained psychiatrists using the Structured Clinical Interview for DSM-IV-TR [35] to confirm the existence of current or past psychiatric disorders. The demographic and clinical information of the patients were also systematically assessed. Patients were excluded if they met the following criteria: psychiatric disorders with psychotic symptoms, other anxiety disorders, substance dependence, mental retardation (as defined by DSM-IV), history of major head trauma, or current major somatic or neurological disorders. For the control group, we recruited 108 healthy subjects (56 men, 52 women) using posters and online advertisements. According to the DSM-IV-TR diagnostic criteria, subjects with a current or previous lifetime history of any psychiatric disorders were excluded from the study. All participants provided written informed consent according to procedures approved by the Severance Hospital Institutional Review Board and all methods conformed to the approved guidelines.

### Measurement of OC symptoms and clinical characteristics

Clinical symptoms were assessed by a trained psychologist using the 10-item Y-BOCS [36] for OC symptom severity and the Montgomery-Åsberg Depression Rating Scale (MADRS) [37] for depression severity. We also used the OCI-R-K [38, 39] to assess the severity of OC symptoms via dimensions including washing, obsessing, hoarding, ordering, checking, and neutralizing.

### Pyrosequencing procedures

**Fig. 2:**
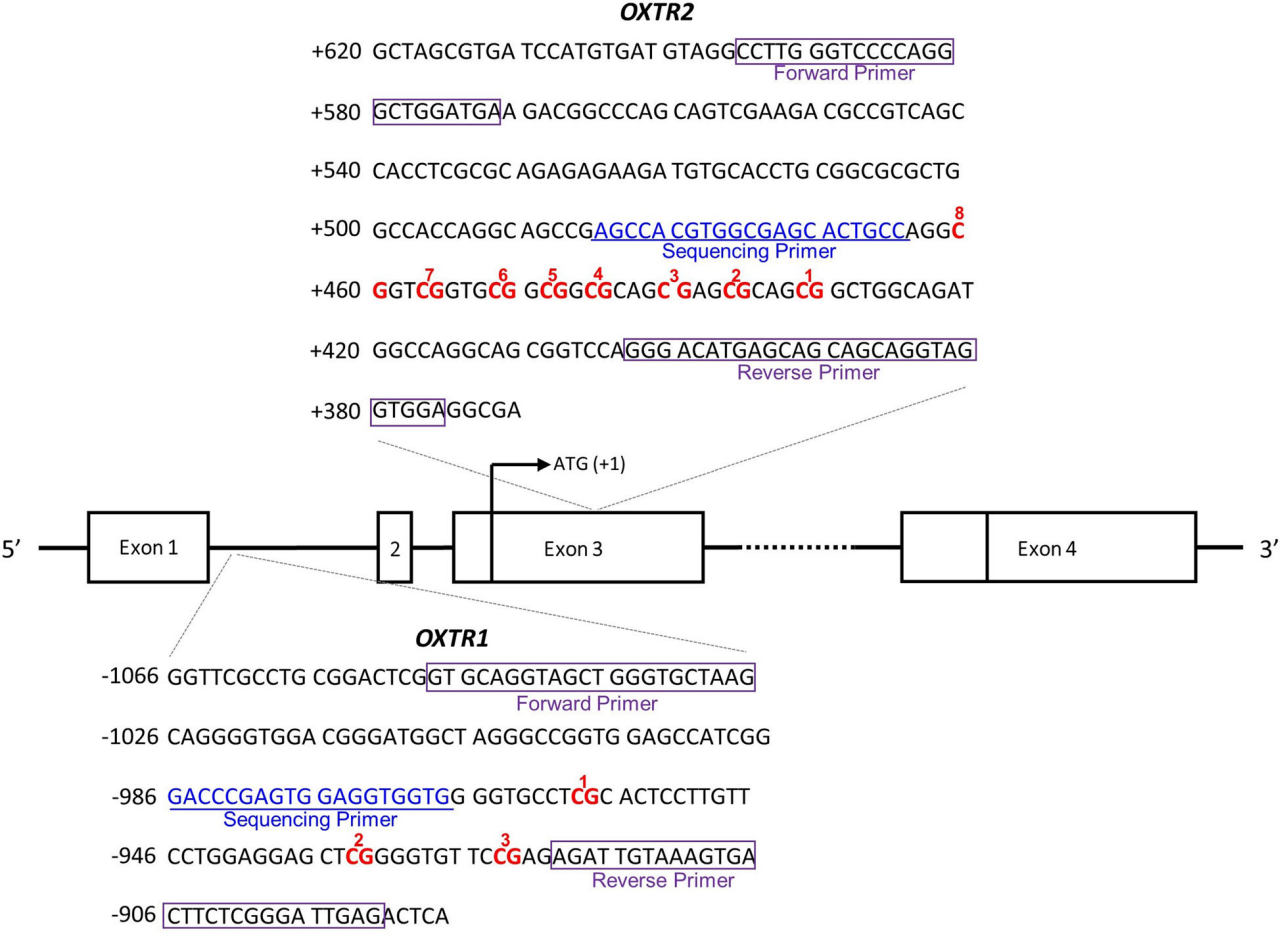
The location of CpG-rich regions in the oxytocin receptor gene and target primer sequences. The sequence is displayed according to GRCh37 build, National Center for Biotechnology Information (NCBI) reference sequence NC_000003.11. CpG sites are numbered (OXTR1, CpG1 = Chr3: 8,810,833, CpG2 = Chr3: 8,810,808, CpG3 = Chr3: 8,810,798; OXTR2, CpG1 = 8,809,413, CpG2 = 8,809,417, CpG3 = 8,809,422, CpG4 = 8,809,425, CpG5 = 8,809,428, CpG6 = 8,809,433, CpG7 = 8,809,437, and CpG8 = 8,809,442)

Three CpG sites in the 5′ UTR of *OXTR* exon 2 (*OXTR1*) and eight CpG sites in the protein coding region of *OXTR* exon 3 (*OXTR2*) were targeted based on previous studies: *OXTR1* (Chr3: 8,810,729–8,810,845; GRCh37/hg19) [30] and *OXTR2* (Chr3: 8,809,281–8,809,534; GRCh37/hg19) [11, 40]. The *OXTR1* included the CpG site -934 (relative to translation start site) in which elevated DNA methylation level was reported to be related to decreased expression of *OXTR* in temporal cortex tissue of autistic males [4], and the methylation level was revealed to be associated with brain activity in dorsal anterior cingulate cortex which is important in OCD pathophysiology [30]. We also targeted CpG sites in *OXTR2* which were reported to have significant relationships with response to social stress in social phobia or non-clinical subjects [11, 40], considering that several studies suggested an impairment of social cognition in patients with OCD [41, 42]. These selected CpG sites are shown in Fig. 2.

Genomic DNA was extracted from whole blood using standard techniques by *DNA Link*, *Inc*. (Seoul, Republic of Korea). The pyrosequencing procedure was used for DNA methylation analysis by *Genomictree*, *Inc*. (Daejeon, Republic of Korea). The bisulfite conversion was conducted using the EZ DNA Methylation-Lightning kit (Zymo Research, CA, USA). The target regions were amplified using polymerase chain reaction (PCR) with converted DNA, and the PCR was conducted in accordance to the general guidelines of pyrosequencing. Successful PCR products were confirmed using electrophoresis on a 2% agarose gel. The pyrosequencing was performed using a PyroMark ID system with the Pyro Gold reagents kit (Qiagen, Hilden, Germany). To control for the internal quality of completeness of bisulfite treatment, the analysis of a non-CpG cytosine was included during pyrosequencing. More detailed information of these procedures have been described elsewhere [12].

### Statistical analysis

All data analysis was conducted using SPSS 25 software (IBM Corp., Armonk, NY, USA). Descriptive statistics were calculated for demographic and clinical variables, with continuous variables presented as the mean ± standard deviation (SD). Differences in the demographic data between patients with OCD and healthy controls were assessed using *t* tests. Data were examined for normality by visual inspection of their histograms and the Shapiro-Wilk test. For variables with non-normal distribution, normal score with the Blom method [43] was used for group comparisons. Since the DNA methylation levels were not normally distributed, Blom transformation was applied. The distributions of the raw data of methylation levels of individual CpGs (Supplementary Fig. 1) and a description of the normalization process are included in the supplementary material.

MANCOVA was conducted to evaluate the influence of OCD status on *OXTR1* and *OXTR2* DNA methylation levels with covariates of age and education level. Given that oxytocin system function is sexually dimorphic [44–46], we conducted the analyses separately for men and women. Since DNA methylation levels at the eight *OXTR2* CpG sites were highly intercorrelated (Cronbach’s alpha, men with OCD 0.99, women with OCD 0.985, control men 0.981, control women 0.975), the average methylation level of the eight CpG sites was used as a variable for the analyses. Thus, we used the DNA methylation levels at four CpG sites (three *OXTR1* CpGs and one averaged value for 8 *OXTR2* CpGs) as dependent variables in the MANCOVA model. Additionally, to rule out potential effects of medication, we compared the DNA methylation status between drug-naïve patients and healthy controls, and drug-naïve and drug-treated patients using MANCOVA. Statistical significance was adjusted for the multiple comparisons of the four CpG sites using the Bonferroni method (*α* = 0.0125).

To investigate the relationships between *OXTR* DNA methylation levels and clinical OCD characteristics as measured by Y-BOCS and OCI-R-K, partial correlation coefficients were calculated, controlling for the potential confounding effects of sex, age, and MADRS score. Partial correlation coefficients were used for drug-naïve patients with OCD (*n* = 45) to exclude the potential effects of medication.

## Supplementary information

**Additional file 1: Supplementary material.** Evaluation of Normality for DNA methylation levels of CpG sites. **Supplementary Fig. 1.** Histograms of the variables of the CpG sites for each group in males and females. **Table S1.** Results of MANCOVA^a^ of DNA methylation at OXTR CpG sites between drug-naïve patients with OCD and drug-treated patients with OCD for men and women. **Table S2.** Partial correlation^a^ between DNA methylation levels at OXTR1 CpG1 and CpG2 and OC symptom dimensions based on OCI-R-K in drug-naïve patients with OCD (*n* = 45)

## Data Availability

Not applicable.

## Abbreviations

*OXTR*: Oxytocin receptor gene
OCD: Obsessive-compulsive disorder
CpG site: Cytosine-phosphate-guanine site
UTR: Untranslated region
ASD: Autism spectrum disorder
MANCOVA: Multivariate analysis of covariance
Y-BOCS: Yale-Brown Obsessive Compulsive Scale
OC symptom: Obsessive-compulsive symptom
MADRS: Montgomery-Åsberg Depression Rating Scale
OCI-R-K: Korean version of Obsessive-Compulsive Inventory-Revised
*OXTR1*: 5′ UTR of OXTR exon 2
*OXTR2*: OXTR exon 3
PCR: Polymerase chain reaction
