# Supplementary material for: Reduced DNA methylation of the oxytocin receptor gene is associated with obsessive-compulsive disorder
Source: Clin Epigenetics. 2020 Jul 6;12:101. doi: 10.1186/s13148-020-00890-w (PMC7336407; doi:10.1186/s13148-020-00890-w)
Supplement: Supplementary file 1 — Additional file 1: Supplementary material. Evaluation of Normality for DNA methylation levels of CpG sites. Supplementary Fig. 1. Histograms of the variables of the CpG sites for each group in males and females. Table S1. Results of MANCOVAa of DNA methylation at OXTR CpG sites between drug-naïve patients with OCD and drug-treated patients with OCD for men and women. Table S2. Partial correlationa between DNA methylation levels at OXTR1 CpG1 and CpG2 and OC symptom dimensions based on OCI-R-K in drug-naïve patients with OCD (n = 45) [file 13148_2020_890_MOESM1_ESM.docx]

**Supplementary material**

<Supplementary Methods>

**Evaluation of Normality for DNA methylation levels of CpG sites**

Normality was evaluated before conducting MANCOVA. When raw data of DNA methylation levels were examined for normality by visual inspection of their histograms and the Shapiro-Wilk test, none of the CpGs was normally distributed, as some skewness and kurtosis were present in each of their distributions (Supplementary Fig. 1). Thus, all variables of the methylation levels were normalized using Blom method in each sex. Normality was again evaluated by visual inspection of their histograms and the Shapiro-Wilk test, and all the variables displaced normal distributions following the transformation.

<Supplementary Results>

**Supplementary Fig. 1.** Histograms of the variables of the CpG sites for each group in males and females


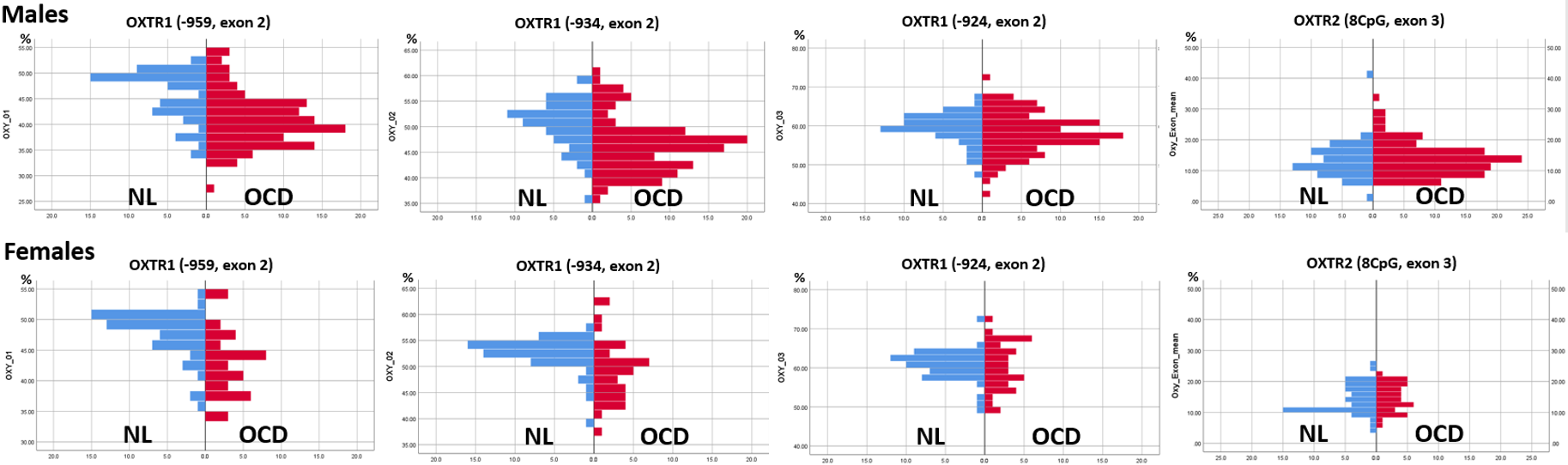


| **Table S1.** Results of MANCOVA^a^ of DNA methylation at OXTR CpG sites between drug-naïve patients with OCD and drug-treated patients with OCD for men and women. | | | | | | | | | | | |
| --- | --- | --- | --- | --- | --- | --- | --- | --- | --- | --- | --- |
|  | | Men | | | | | Women | | | | |
|  |  | Drug-naïve^b^ (n = 32) | Drug-treated^b^ (n = 80) |  | | | Drug-naïve^b^ (n = 13) | Drug-treated^b^ (n = 26) |  | | |
|  |  |  |  | F | P^c^ | η_p_^2^ |  |  | F | P^c^ | η_p_^2^ |
|  |  | Statistics: Wilks λ = 0.972, F(4, 105) = 0.744, p = 0.564 | | | | | Statistics: Wilks λ = 0.938, F(4, 32) = 0.528, p = 0.716 | | | | |
| ***OXTR*1 (exon2)** | CpG1  (-959) | 41.49 ± 5.66 | 40.72 ± 4.99 | 0.250 | 0.618 | 0.002 | 42.75 ± 5.96 | 43.04 ± 4.78 | 0.045 | 0.833 | 0.001 |
|  | CpG2  (-934) | 46.41 ± 4.73 | 46.29 ± 5.42 | 0.085 | 0.771 | 0.001 | 48.87 ± 3.72 | 49.23 ± 6.60 | 0.075 | 0.786 | 0.002 |
|  | CpG3  (-924) | 57.41 ± 4.45 | 58.35 ± 5.53 | 1.524 | 0.220 | 0.014 | 61.49 ± 6.12 | 59.80 ± 5.77 | 0.562 | 0.459 | 0.016 |
| ***OXTR*2 (exon3)** | Mean value of CpG1-8 | 14.32 ± 6.31 | 13.60 ± 5.11 | 0.363 | 0.548 | 0.003 | 13.79 ± 4.27 | 15.31 ± 4.59 | 0.544 | 0.466 | 0.015 |
| MANCOVA, Multivariate analysis of covariance; OCD, obsessive-compulsive disorder; HC, healthy control | | | | | | | |  |  |  |  |
| ^a^MANCOVA demonstrated significant between-group difference after controlling for age and education. | | | | | | | |  |  |  |  |
| ^b^Mean ± standard deviation of raw data | | | | | | | | | | | |
| ^c^Statistical significance was set at P < 0.0125 after Bonferroni correction for 4 CpG sites | | | | | | |  |  |  |  |  |

**Table S2.** Partial correlation^a^ between DNA methylation levels at *OXTR1* CpG1 and CpG2 and OC symptom dimensions based on OCI-R-K in drug-naïve patients with OCD (n = 45)

| **OCI-R-K** | **CpG1** | **CpG2** |
| --- | --- | --- |
| Washing | -0.22 | 0.046 |
| Obsessing | -0.079 | -0.265 |
| Hoarding | 0.019 | -0.05 |
| Ordering | -0.422* | 0.031 |
| Checking | -0.029 | -0.17 |
| Neutralizing | -0.1 | -0.028 |
| OCI-R-K, obsessive-compulsive inventory-revised-Korean version; OCD, obsessive-compulsive disorder | | |
| ^*^p= 0.005 |  |  |
| ^a^Sex, age, and MADRS score are controlled. | | |
